# The Mediating Roles of Core Self-Evaluation and Career Exploration in the Association Between Proactive Personality and Job Search Clarity

**DOI:** 10.3389/fpsyg.2021.609050

**Published:** 2021-06-04

**Authors:** Hongrui Zhu, Hua Zhang, Aixian Tu, Siqi Zhang

**Affiliations:** ^1^School of International Nursing, Hainan Medical University, Haikou, China; ^2^School of Management, Hainan Medical University, Haikou, China

**Keywords:** proactive personality, core self-evaluation, career exploration, job search clarity, mediating effect

## Abstract

In recent years, university students’ employment has become an increasingly prominent problem worldwide. Improving the job search clarity of students is a great way to boost job-hunting success. Proactive personality may predict job search clarity through the mediating effects of core self-evaluation and career exploration. However, few studies have explored this relationship and the mediating roles of core self-evaluation and career exploration. To identify the relationship between a proactive personality and job search clarity and the mediating roles of core self-evaluation and career exploration, a cross-sectional survey was conducted. A total of 495 students majoring in nursing completed the questionnaire which consisted of the proactive personality scale, core self-evaluation scale, career exploration survey, and job search clarity scale. Correlation analyses and mediation analyses were conducted using SPSS 24.0 and AMOS 24.0 respectively. This study showed that students higher on the proactive personality scale were more likely to perform better on job search clarity. Core self-evaluation and career exploration have fully mediating effects on the relationship between a proactive personality and job search clarity. Therefore, interventions concentrated on cultivating proactive personality, improving core self-evaluation, and strengthening career exploration would be necessary for increasing job search clarity.

## Introduction

In recent years, employment for university students has become an increasingly prominent problem worldwide and a hot topic for education research. With the enrollment expansion of nursing students in the bachelor’s degree program, nursing undergraduates also faced employment problems. Employment issues are not only important for the lives of millions of households but also directly related to social stability. Previous studies have found that job search clarity is positively related to job-hunting behavior and job-hunting results. Therefore, one of the great ways to solve the employment problem of university students is to improve students’ job search clarity to boost their job-hunting success.

Job search clarity is defined as “the extent to which job seekers have clear job search objectives and have a clear idea of the type of career, work, or job desired” (Wanberg et al., 2002). Cote believed that the definition of job search clarity should also include (a) how to find a job and (b) how long it takes to find a job (Cote et al., 2006). The concept of job search clarity is derived from goal-setting theory. In the job-hunting process, job search clarity plays a goal-oriented role, which has an important influence on job search behavior and outcomes (Wang, 2016; Wang and Dai, 2017). Job seekers with higher job search clarity tend to have a clearer idea of the type of job they desire and how to search for a job. They engage in more search behaviors to achieve clear goals, which in turn lead to more job interviews and more job offers. However, job seekers who lack clarity spend time on exploring different options and search less intensely, as a result, receiving fewer job interviews and job opportunities. Besides, Wanberg et al. (2002) found that job search density plays a mediating role in the relationship between job search clarity and job search outcome. From the literature review, we find that the studies mainly focused on the positive effects of job search clarity on job search behavior, job search density, and job search outcome. But few focused on the antecedent factors. Job search clarity is critical to career development and success. Job search clarity can be improved with appropriate coaching and training. Therefore, this study will explore the antecedent variables of job search clarity and the mediating mechanism with a new perspective. It will be meaningful for deepening the career development theory and career success theory and building a research framework for follow-up studies. It can also provide theoretical support for improving job search clarity of nursing undergraduates and other majors students so as to promote students’ career development and vocational counseling work among universities. This will optimize students’ job-seeking behavior and outcomes so that the unemployment problem of college students could be ultimately solved.

### Theory and Hypotheses

Job search clarity is an important component of Social Cognitive Career Theory (SCCT). According to SCCT, individual, environmental, and behavioral variables can influence individual career development (including career interests, career goals, and career values) through interaction (Lent and Brown, 2011). Therefore, individual, environmental, and behavioral factors can be considered as the antecedent variables of job search clarity. Among the three factors, only the individual and behavioral factors can be self-controlled. Therefore, this study aimed to discuss how the individual and behavioral factors affect job search clarity.

### Proactive Personality and Job Search Clarity

Among the individual factors influencing job search clarity, scholars stressed the important impact of personality factors on job search clarity. Compared with the Big Five personality factors, proactive personality has a stronger predictive effect on career development (Major et al., 2006). Furthermore, job search clarity is an important part of career development. Besides, proactive personality is defined as “active attempts made by the individual to effect changes in his or her environment” (Zampetakis, 2008). Job search clarity is individual cognition about the career development goals. According to Cognitive Development Theory, proactive personality plays a key role in the process of cognitive development by driving individual learning and development. Therefore, the formation and development of job search clarity can also be positively affected by proactive personality. Crant and Bateman’s research showed that individuals with proactive personality are able to set effective and change-oriented goals (Crant and Bateman, 2000). Shen and Hu found that individuals with stronger proactive personality are more likely to explore themselves and the external environment actively, and they tend to have clearer job-hunting intentions and career development goals (Shen and Hu, 2015). Li also found that among a variety of individual factors, proactive personality has the strongest impact on job search clarity (Li, 2019).

Therefore, we propose Hypothesis 1:

Hypothesis 1 (H1): Proactive personality is positively related to job search clarity.

### The Mediating Role of Career Exploration

Guan et al. proposed that the antecedent variables, which belong to the same domain as the outcome variables, tend to have higher predictive power for the outcome variables (Guan et al., 2015). Career exploration and job search clarity both belong to the professional domain, so career exploration is selected as the behavioral variable affecting job search clarity. The career exploration process is a series of career-related behaviors for people to explore themselves and/or the environment (Lee et al., 2016). According to Super’s theory, career development consists of five stages and university students are in the career exploratory stage, the most important period in the development of an individual (Super, 1990). The career exploratory stage can be subdivided into experimental stage, transitional stage, and trial stage. University students are mainly in the experimental stage, which means that individuals begin to comprehensively understand their interests, abilities, and professional social values, and then decide their careers. Although the professional direction has been set for nursing undergraduates, they still have great difficulties in job-searching decisions. Especially in China, many nursing undergraduates are transferred to nursing major unwillingly. They know little about nursing before studying this major. However, through several years’ study in university, students can constantly explore themselves and the external environment by studying nursing courses, getting value guidance from teachers, taking part in professional development activities, participating in cadet and internships, and so on, which is helpful for them to identify their own personal characteristics (including interests, abilities, etc.), get information about occupations (professional social values, job opportunities, etc.) and set clear career goals ultimately.

In addition, according to the Active Behavior Theory proposed by Crant and Bateman, individuals with stronger proactive personality will be more likely to adapt to, control, and even create the surrounding environment through active attempts and a series of behaviors (Crant and Bateman, 2000). The series of behaviors include actively searching for information, seeking feedback, and so on. While career exploration also includes these behaviors. Therefore, it is speculated that proactive personality promotes career exploration behavior. In addition, the relationship between proactive personality and career exploration has also been tested by some empirical studies. Cai et al. (2015) and Qu et al. (2015) proved that there was a significant positive correlation between proactive personality and career exploration.

To sum up, in the process to form and construct job search clarity, the individuals with stronger proactive personality will be more active in career exploration, including actively collecting information about themselves and their occupational environment. These behaviors will help them to achieve clear job goals gradually. Therefore, we hypothesize the following:

Hypothesis 2 (H2): Career exploration plays a mediating role between proactive personality and job search clarity.

### The Mediating Role of Core Self-Evaluation

Social Cognitive Career Theory also proposes that self-efficacy can act on individuals’ career goals and affect their job-hunting, career selection, and career development process (Lent and Brown, 2011). Ye’s study indicated that college students’ job search clarity is influenced by their sense of self-efficacy (Ye et al., 2017). Core self-evaluation (CSE) is an individual variable, which has a similar function with self-efficacy. So it is also worth exploring the relationship between CSE and job search clarity. CSE is defined as a high-order personality, including four personality traits: locus of control, general self-efficiency, self-esteem, and neuroticism, which involves an individual’s baseline evaluation of his ability and value (Xiao et al., 2014; Barać et al., 2018). Students with a higher score of CSE, embodied with the internal control personality traits and a higher level of self-identification and acceptance, tend to have a deeper understanding of themselves, which are conducive to them to make more suitable career choices. Besides, students with stronger self-esteem and self-efficacy are always more confident in their own abilities and career prospects. They are more likely to have clear career goals (Huang and Xu, 2014) Erez and Judge discovered that CSE is associated with job outcomes and that the mediator is the job search goal setting (Erez, and Judge, 2001). In addition, according to Seibert’s theory, CSE as a cognitive variable has a mediating function between proactive personality and career success. Individuals with high initiative always hold positive cognition of their own abilities and are more likely to show high core self-evaluation, manifested in high self-efficacy, high self-esteem, stable moods, and a healthy locus of control (Hsieh and Huang, 2014). Given these rationales, we propose Hypothesis 3:

Hypothesis 3 (H3): Core self-evaluation plays a mediating role between proactive personality and job search clarity.

### The Chain-Mediating Effect of Core Self-Evaluation and Career Exploration

According to the three-level variable model proposed by Matthews et al. (2006), if the variable at the first level is trait construct (such as personality characteristic), its influence on the outcome variable (such as problem behavior) at the third level often needs to be realized through the mediating variable at the second level (Matthews et al., 2006). Based on this model, the effect of proactive personality, as a kind of personality characteristics, on career exploration behavior needs to be realized by some intermediary variables. Core self-evaluation, as a self-adjusting variable about self-assessment, is worth exploring as the mediating role between proactive personality and career exploration. In the process of career exploration, proactive personality can stimulate the internal self-verification mechanism of core self-evaluation, and promote the interaction between core self-evaluation and career exploration. In recent years, some studies have been carried out to explore the relationship between core self-evaluation and career exploration (Zhu and Chang, 2021). Individuals with higher core self-evaluation tend to have more positive self-concepts about themselves and feel more confident in their abilities, so that they are more likely to generate motivation to take part in the activities of career exploration. In addition, individuals with higher core self-evaluation will conduct more career exploration activities to verify themselves. Therefore, core self-evaluation is positively related to career exploration. Besides, proactive personality can influence core self-evaluation positively and career exploration can predict job search clarity. Therefore, we put forward the following hypothesis:

Hypothesis 4 (H4): Core self-evaluation and career exploration play chain-mediated roles between proactive personality and job search clarity.

**Figure 1:**
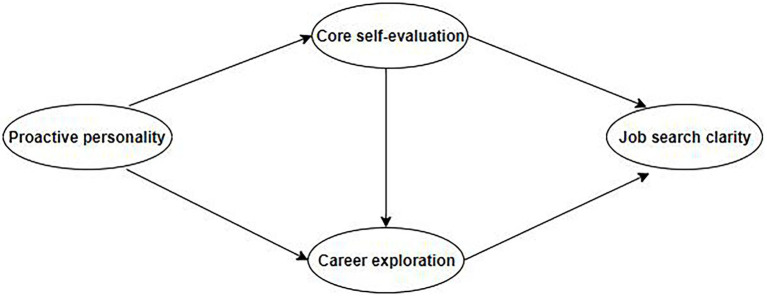
The Hypothesized model.

In conclusion, based on the hypotheses above, this study constructed a multi-mediation model. The operationalization of the mediation model in the current study is described in Figure 1.

## Materials and Methods

### Participants

Cluster sampling was used in this study. The data were collected from nursing undergraduates at one university which was the only university having bachelor of science in nursing program in Hainan. All the participants voluntarily participated in this study and provided written informed consent before completing the questionnaires. A total of 533 questionnaires were handed out, and 38 surveys were eliminated because some participant responses were incomplete or not true. This ultimately resulted in 495 valid questionnaires, which consisted of 73 questionnaires from men and 422 questionnaires from women. The ages of the students ranged from 17 to 25 with a mean of 20.68 (*SD* = 1.448).

### Procedures

The questionnaires were handed out to all nursing undergraduates at one university in Hainan in June 2020. Participants completed the questionnaires in classroom. Before completing the questionnaires, the research’s aim and content were informed and written informed consent was obtained from every participant. The questionnaire instructions were explained to the participants in detail by investigators who had received unified training. All the participants were asked to read the questionnaire carefully and answer the questions according to their actual situations. The questionnaire took about 20 min to complete.

### Instruments

#### Proactive Personality Scale

The PPS was developed by Crant and Bateman. The Chinese version of the PPS, translated and modified by Shang and Gan, was a one-dimensional scale, including 11 items rated from 1 (strongly disagree) to 7 (strongly agree). The Cronbach’s alpha coefficient of this scale was 0.86 (Shang and Gan, 2009).

#### Core Self-Evaluation Scale

This scale was a self-reported measure of CSE, including 12 items, and developed by Judge and colleagues. Based on differences in culture and expression habits between Chinese and Westerners, Du translated and adjusted the scale into a Chinese version. Two items of the original scale were deleted, namely, A3 (as long as I work hard, I will generally succeed) and A9 (I am able to decide what will happen in my life). The respondents rated the items from 1 (strongly disagree) to 5 (strongly agree). The total score was between 10 and 50. A higher score indicated a higher level of self-evaluation. Cronbach’s alpha coefficient of CSES was 0.83 and the split-half reliability was 0.84, and the test-retest reliability coefficient was 0.82 for the entire scale (Du et al., 2012).

#### Job Search Clarity

This 5-item scale was developed by Cote. The items were rated from 1 (strongly disagree) to 5 (strongly agree). The total score ranged from 5 to 25. Higher the score stated greater clarity of job search (Cote et al., 2006). Based on differences in culture and expression habits between Chinese and Westerners, the scale was translated and adjusted into a Chinese version. Cronbach’s alpha coefficient of the scale (Chinese version) was 0.82 (Ye et al., 2017).

#### Career Exploration Scale

The CES was developed by Stumpf. And the scale was translated and adjusted into a Chinese version with 18 items. This 18-item scale included four dimensions: environment exploration (1–4), self-exploration (5–9), system exploration (10–15), and information quantity (16–18). The items were rated from 1 (very few) to 5 (very many). A higher score represents higher motivation to explore. The internal consistency reliability of the scale was 0.88. And the Cronbach’s alpha coefficients for the four dimensions of CES were 0.84, 0.76, 0.74, and 0.75, respectively (Xu, 2008).

### Data Analysis

In this study, SPSS 24.0 was used for statistical analysis, including statistical description and correlation analysis. In addition, AMOS 24.0 was utilized to construct the models and conduct mediating effect analyses.

The variables were described as the mean and standard deviation (SD). In addition, Pearson’s correlation analysis was used appropriately to examine the correlations between proactive personality, core self-evaluation, career exploration, and job search clarity.

To construct a superior model, the variables of proactive personality and core self-evaluation were both created into two item parcels by utilizing the item-to-construct balance technique because both the proactive personality scale and the core self-evaluation scale have only a single dimension. Some scholars believed that item parceling is very useful for building an SEM framework (Little et al., 2002). It has been verified that parceling items in a scale or subscale into several small parts of items has important significance in improving the variable-to-sample size ratio and increasing the stability of parameter estimates (Tian et al., 2018).

Then, to test the structural model, the maximum-likelihood estimation in the AMOS 24.0 program was used. We estimated the fit of each model to the data. The following five indices were used to evaluate the goodness of fit of the model: (1) the chi-square statistic (*χ*^2^) and the chi-square-to-degrees-of-freedom ratio (*χ*^2^/*df*) (2) the root mean square error of approximation (RMSEA) (3) the goodness-of-fit index (GFI) (4) the comparative fit index (CFI), and (5) the Tucker-Lewis Index (TLI). In this study, a model with a good fit was determined by the fitting measures meeting the following criteria: *χ*^2^/*df* < 3; GFI, CFI, and TLI ≥ 0.90 (the closer to 1, the better the index); and RMSEA ≤ 0.08 (the closer to 0, the better the RMSEA; Kim et al., 2009). Next, the research hypotheses were tested by examining whether each structural path was statistically significant, and then the non-significant paths were removed to form the optimal mediation model. Finally, bootstrapping procedures were used to further confirm the mediation effects. A total of 5,000 bootstrapped samples were drawn, and 95% confidence intervals (CIs) with bias corrections were reported in this analysis. Statistical significance was determined with a 95% CI that did not contain zero (Hayes, 2012).

## Results

### Descriptive Statistics and Correlation Analysis

Table 1 presents the means, standard deviations, and interrelations of all the variables. These results demonstrated that a proactive personality was significantly related to core self-evaluations (*r* = 0.410, *p* < 0.01) and career exploration (*r* = 0.490, *p* < 0.01), which indicated that nursing undergraduates with a proactive personality tended to have higher levels of core self-evaluation and career exploring behaviors. Both core self-evaluation scores and career exploration scores were significantly correlated with job search clarity (*r* = 0.381 and 0.377, respectively; *p* < 0.01), which meant that students with higher scores on the core self-evaluation scale and career exploration scale had better job search clarity. In addition, the core self-evaluation score was positively associated with the career exploration score (*r* = 0.334, *p* < 0.01), which indicated that students with a higher score on the core self-evaluation scale were found to perform better on career exploration.

**Table 1:** Means, standard deviations (SD) and correlations of all variables.

Variables	Mean	*SD*	1	2	3	4
1.Proactive personality	54.24	9.67	—			
2.Core self-evaluation	33.27	5.45	0.41^**^	—		
3.Career exploration	54.63	10.91	0.49^**^	0.33^**^	—	
4.Job search clarity	16.60	6.22	0.30^**^	0.38^**^	0.38^**^	—

^**^p < 0.01.

### Structural Model

To test the hypotheses and the proposed research model (shown in Figure 1), a structural equation analysis of the correlations between a proactive personality, core self-evaluation, career exploration, and job search clarity was conducted. The mediation model was established. The fit indices for Model 1 were acceptable: chi-squared = 49.164, *df* = 18, *χ*^2^/*df* = 2.731, *p* < 0.001, RMSEA = 0.059, GFI = 0.980, CFI = 0.986, and TLI = 0.971 (shown in Table 2). However, the standardized direct path coefficient from a proactive personality to job search clarity was not statistically significant (*β* = 0.014, *p* > 0.05).

**Table 2:** Comparison of structural equation models (Model 1 and Model 2).

Model	*x*^2^	*df*	△*x*^2^(△*df*)	RMESA	GFI	CFI	TLI
Model 1	49.16^***^	18	2.73	0.06	0.98	0.98	0.97
Model 2	49.22^***^	19	2.59	0.05	0.98	0.98	0.97

*N* = 495; *x*^2^: Chi-square statistic.

Model 1: proactive personality→core self-evaluation + career exploration→ job search clarity (additional line: proactive personality →job search clarity).

Model 2: proactive personality→core self-evaluation + career exploration →job search clarity.

*** *p* < 0.001.

**Figure 2:**
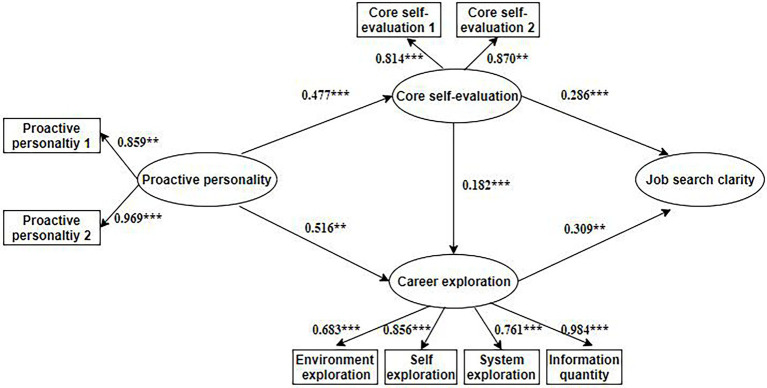
The final model with standardized estimates (*N* = 495). ^**^p < 0.01, ^***^p < 0.001.

Next, the insignificant direct path was deleted from Model 1 to build Model 2 (Figure 2). Table 2 indicates that Model 2 had a better fit to the data (*χ*^2^ = 49.224, *df* = 19, *χ*^2^/*df* = 2.591, *p* < 0.001, RMSEA = 0.057, GFI = 0.980, CFI = 0.986, and TLI = 0.973). In addition, Figure 2 shows that all the path coefficients are statistically significant. Proactive personality was significantly and positively correlated with core self-evaluation and career exploration (*β* = 0.477, *p* < 0.001; and *β* = 0.516, *p* < 0.01). The standardized direct path coefficients from core self-evaluation and from career exploration to job search clarity were also statistically significant (*β* = 0.286, *p* < 0.001 and *β* = 0.309, *p* < 0.01, respectively). Additionally, core self-evaluation was also significantly related to career exploration (*β* = 0.182, *p* < 0.001). Moreover, compared with Model 1, Model 2 was more parsimonious. Based on the theory of Kelloway, if two competing models have similar results, the parsimonious model should be adopted (Kelloway, 1998). Therefore, Model 2 was adopted in this study (Insert Table 2 and Figure 2).

### Bootstrap Result of the Mediating Effect

The significance of the mediating effects of core self-evaluation and career exploration between proactive personality and job search clarity was tested using the bootstrap estimation procedure in AMOS (a bootstrap sample of 5,000 was specified; Preacher et al., 2007). Table 3 presents that the indirect effects of a proactive personality on job search clarity through core self-evaluation and career exploration were both significant (empirical 95% confidence intervals did not contain zero).

**Table 3:** Bootstrapping regression results for proactive personality mediated by core self-evaluation and career exploration. (*N* = 495).

Variable	Estimate	Lower 2.5%	Upper 2.5%	*P*
Proactive personality →Core	0.14	0.07	0.22	0.000
self-evaluation →Job search clarity				
Proactive personality →Career	0.16	0.09	0.24	0.000
exploration →Job search clarity				
Proactive personality →Core	0.03	0.01	0.07	0.000
self-evaluation →Career				
exploration → Job search clarity				

## Discussion

The several hypotheses were all verified. Proactive personality was positively correlated with job search clarity. And core self-efficacy and career exploration played simple mediating roles and chain mediating roles in the relationship between proactive personality and job search clarity. These findings were significant for developing related career theory and proposing intervention strategies to improve job search clarity.

### Theoretical Implications

Most of the previous studies on job search clarity focused on exploring how job search clarity predicts career outcomes. Few studies have been conducted on the antecedents of job search clarity. This study proposed a mediation model, including a proactive personality (independent variable), job search clarity (dependent variable), and core self-evaluation and career exploration (mediating variables), which filled the gap in this research field and provided evidence of how a proactive personality predicted job search clarity. The results showed that individuals who scored higher in proactive personality were more likely to have better job search clarity. This finding regarding the positive significance of proactive personality in the personal career development process is consistent with the finding reported in a previous study (Hu et al., 2018). The mediation model also provides a useful and holistic view to understand how a proactive personality relates to job search clarity. This can be ascribed to the contribution made by core self-evaluation and career exploration through mediating processes and mechanisms.

The empirical results showed that core self-evaluation played an intermediary role between a proactive personality and job search clarity. This suggests that individuals with higher proactive personality scores tend to have higher levels of core self-evaluation. The students who scored high on core self-evaluation always felt confident in their personal abilities and were capable of setting clear job goals through repeated practice. This study not only reconfirms Erez and Judge’s result that core self-evaluation is positively correlated with job search clarity, but it also extends the current literature on core self-evaluation. Previous studies have mainly concentrated on the relationships with career satisfaction (Barać et al., 2018), job burnout (Li et al., 2014), work engagement (Tims and Akkermans, 2017), and workplace well-being (Lin et al., 2017). Moreover, core self-evaluation was rarely considered as a whole in these studies. The majority of the literature has studied the four traits of core self-evaluation separately (Pérez-Fuentes et al., 2018; Chen et al., 2019).

Our data also indicated that career exploration played a mediating role in the relationship between a proactive personality and job search clarity. Students high on the proactive personality scale are more likely to take part in more career exploration activities and have a clearer understanding of their careers. This result further revealed the mechanism of the effect of proactive personality on job search clarity. In addition, it also confirmed the career development theory of Super (Super, 1990), highlighted the significant predictive function of career exploration on individual career development (Zikic and Klehe, 2006; Cai et al., 2015), and enriched the literature on the effect of career exploration on job search clarity.

Another important finding was that apart from the simple mediating roles, core self-evaluation and career exploration also played chain-mediated roles between proactive personality and job search clarity. Core self-evaluation was positively and significantly associated with career exploration. Students higher in core self-evaluation are more likely to be curious about the surrounding environment, study with enthusiasm, and have a great spirit of exploration. Therefore, this kind of individual tends to actively engage in more career exploration. Consistent with Taber’s study (Taber and Blankemeyer, 2015), this finding is noteworthy in confirming the importance of core self-evaluation and its related mechanisms. It also verifies that cognitive variables have important significance in developing theories related to career exploration behavior, which contributes to the field of career exploration research.

### Practical Implications

The findings of this study have significant practical implications for improving students’ job search clarity and promoting career development, which are mainly embodied in the following three aspects.

First, our empirical results showed that proactive personality affected core self-evaluation and career exploration and further promotes job search clarity. Therefore, it is necessary to cultivate students’ proactive personality. Psychology courses should be added to the traditional curriculum, and psychological training should be strengthened. This is beneficial for students to analyze their own personality traits and build excellent psychosocial quality. Furthermore, teachers should guide students to consciously make full use of their psychological knowledge to learn how to regulate emotions and foster a positive mental attitude. This will help students become more proactive in life and their studies. In addition, universities should provide development platforms for students and encourage all students to participate in a variety of activities, such as social practice, featured cultural performances, calligraphy competitions, and other activities. These correspondingly reinforce the initiative of students.

Second, given that core self-evaluation positively affects career exploration and job search clarity, it is necessary to improve students’ core self-evaluation levels. In light of the positive influence of a proactive personality on core self-evaluation, measures to develop a proactive personality can be used to enhance self-evaluation. Moreover, universities should also establish psychological consultation centers and popularize psychological health knowledge for students to relieve emotions after experiencing negative comments. For example, psychology teachers can help students reduce the anxiety caused by study stress through relaxation and mindfulness training. This can enhance the students’ self-control, anti-frustration ability, self-confidence, and positive self-statements.

Third, the results also indicated that career exploration has a positive impact on job search clarity. Hence, some potential career guidance and management interventions should be taken. It is important that universities organize professional vocational guidance teachers and establish professional guidance courses to effectively guide students to develop career goals and plans. Furthermore, various employment information, including part-time jobs, full-time jobs, internship opportunities, and other information, should be released to students in multiple ways. This will provide opportunities for students to accumulate job-hunting experience and determine the direction of their own career development. In addition, professional teachers should provide students with vocational skills and development training opportunities to improve their professional capacity and confidence. In addition, communicating with teachers timely is crucial for students to achieve a clear career intention when they feel confused during career-related activities.

### Limitations

Although this research contributes to determining the mechanisms of the relationship between proactive personality and job search clarity and the mediating effects of core self-evaluation and career exploration in that relationship, it has several limitations. First, because of the limited scope of the sample, the sample was not representative enough. Future research is required for students majoring in different professionals, from different universities, and from different cultural backgrounds to generalize the findings. Second, a cross-sectional design was utilized in this study. This design rendered it difficult to make causal inferences. Therefore, in future studies, longitudinal designs should be adopted to better understand the process of how proactive personality influences job search clarity. Third, social desirability bias might exist since all the data in the present study were self-reported by nursing students. In future studies, multiple assessment methods should be used to improve the data quality and thereby improve the validity of the results.

## Conclusion

The present research showed that proactive personality could predict job search clarity among undergraduate nursing students, and the results indicated that students with higher proactive personality scores tend to perform better in job search clarity. More importantly, core self-evaluation and career exploration were two important factors that fully mediated the relationship between a proactive personality and job search clarity. Therefore, interventions concentrated on cultivating a proactive personality, improving core self-evaluation, and strengthening career exploration would be meaningful to increase job search clarity.

## Data Availability Statement

The raw data supporting the conclusions of this article will be made available by the authors, without undue reservation.

## Ethics Statement

The studies involving human participants were reviewed and approved by Research Ethics Committee of Hainan Medical University. The patients/participants provided their written informed consent to participate in this study.

## Author Contributions

HZu, HZa, AT, and SZ conducted this study and wrote this paper. All authors contributed to the article and approved the submitted version.

### Conflict of Interest

The authors declare that the research was conducted in the absence of any commercial or financial relationships that could be construed as a potential conflict of interest.
